# Comparison of disease progression subgroups in idiopathic pulmonary fibrosis

**DOI:** 10.1186/s12890-019-0996-2

**Published:** 2019-11-29

**Authors:** Miia Kärkkäinen, Hannu-Pekka Kettunen, Hanna Nurmi, Tuomas Selander, Minna Purokivi, Riitta Kaarteenaho

**Affiliations:** 1 Division of Respiratory Medicine, Institute of Clinical Medicine, School of Medicine, Faculty of Health Sciences, University of Eastern Finland, P.O. Box 1627, 70211 Kuopio, Finland; 2 Kuopio City Home Care, Rehabilitation and Medical Services for Elderly, Tulliportinkatu 37E, 70100 Kuopio, Finland; 3 Kuopio University Hospital, P.O. Box 100, Puijonlaaksontie 2, 70210 Kuopio, Finland; 4 Department of Clinical Radiology, Kuopio University Hospital, P.O. Box 100, 70029 KYS Kuopio, Finland; 5 Center of Medicine and Clinical Research, Division of Respiratory Medicine, Kuopio University Hospital, P.O. Box 100, 70029 KYS Kuopio, Finland; 6 Science Services Center, Kuopio University Hospital, P.O. Box 100, 70029 KYS Kuopio, Finland; 7 Respiratory Medicine, Research Unit of Internal Medicine, University of Oulu and Medical Research Center Oulu, Oulu University Hospital, P.O. Box 20, 90029 Oulu, Finland

## Abstract

**Background:**

Idiopathic pulmonary fibrosis (IPF) is a progressive interstitial pneumonia with an unpredictable course. The aims of this study were to retrospectively re-evaluate a cohort of patients with IPF according to the 2011 international IPF guidelines and 1) to characterize the subgroups of patients when classified according to their observed survival times and 2) to evaluate whether Composite Physiologic Index (CPI), Gender-Age-Physiology (GAP) Index or clinical variables could predict mortality.

**Methods:**

Retrospective data was collected and patients were classified into subgroups according to their observed lifespans. Differences in clinical variables, CPI and GAP stages as well as in comorbidities were investigated between the subgroups. Predictors of mortality were identified by COX proportional hazard analyses.

**Results:**

A total of 132 patients were included in this study. The disease course was rapid (≤ 2 years) in 30.0%, moderate (2–5 years) in 28.0% and slow (≥ 5 years) in 29.0% of the patients. Pulmonary function tests (PFT) and CPI at baseline differentiated significantly between the rapid disease course group and those patients with longer survival times. However, the predictive accuracy of the investigated clinical variables was mainly less than 0.80. The proportions of patients with comorbidities did not differ between the subgroups, but more patients with a rapid disease course were diagnosed with heart failure after the diagnosis of IPF. Most patients with a rapid disease course were categorized in GAP stages I and II, but all patients in GAP stage III had a rapid disease course. The best predictive multivariable model included age, gender and CPI. GAP staging had slightly better accuracy (0.67) than CPI (0.64) in predicting 2-year mortality.

**Conclusions:**

Although the patients with a rapid disease course could be differentiated at baseline in terms of PFT and CPI, the predictive accuracy of any single clinical variable as well as CPI and GAP remained low. GAP staging was unable to identify the majority of patients with a rapid disease progression. It is challenging to predict disease progression and mortality in IPF even with risk prediction models.

## Background

The clinical course of disease in idiopathic pulmonary fibrosis (IPF) is variable and difficult to predict. It has been estimated that 25% of the patients will live over 5 years after diagnosis; the median survival in several studies has been 2–3 years after diagnosis [1]. Approximately 15–20% of the patients experience acute exacerbations that are usually severe and can be lethal [2]. It is difficult to predict the optimal time for initiating therapeutic treatment, palliative care and lung transplantation, not only because of the absence of an accurate and generally accepted staging system, but also due to the unpredictable course of disease.

Recently, the Composite Physiologic Index (CPI) and the Gender-Age-Physiology (GAP) index have been most commonly applied for estimating the survival of patients with IPF [3–6]. CPI quantifies pulmonary function impairment due to pulmonary fibrosis, which is then correlated with the extent of fibrosis in computed tomography while excluding emphysema [3]. The GAP index classifies patients into three different disease stages and estimates the mortality of the stages at 1-, 2- and 3-years. Some researchers have utilized simultaneously GAP and CPI in their study protocols, the results of which are presented in Table 1 [5–13].

**Table 1:** Studies in idiopathic pulmonary fibrosis utilizing Gender-Age-Physiology (GAP) index and Composite physiologic index (CPI) in their study protocols

Study	Aims and utilization of GAP and CPI	Results and conclusions
[5]	Comparison of GAP and CPI	CPI and GAP were predictive of 1-, 2- and 3-year mortality
[6]	FVC > 80% in comparison with GAP I and CPI ≤40	CPI and GAP significantly predicted disease progression and 1-, 2- and 3-year mortality. Higher scores in GAP (>I) and CPI (> 40) predicted poorer survival.
[7]	Clinical characteristics according to GAP stage	Significant CPI and survival differences between GAP stages
[8]	GAP and CPI in different smoking status groups	CPI was higher in never-smokers. GAP predicted AEx in never-smokers. GAP was strong predictor of mortality in never-smoking patients
[9]	Risk factors of AEx-IPF	GAP and CPI did not differ significantly between AEx and non-AEX groups. GAP stage ≥II was related to survival and risk for AEx. GAP stage ≥ II predicted AEx
[10]	CPI, GAP, DSP and duBois score in survival prediction	All the indexes were predictive for survival, CPI being the most accurate.
[11]	Stratification for CT algorithm and GAP/CPI	CPI estimated severity of ILD better than GAP. CPI was more accurate mortality predictor than GAP. Mortality prediction was improved compared to GAP when stratification was based on CT algorithm and CPI.
[12]	CPI and GAP in mortality prediction during lung transplant assessment	Higher CPI and GAP were associated with mortality
[13]	GAP and CPI in severity assessment in different HRCT patterns	CPI, but not GAP differentiated significantly between the groups

*GAP* Gender-age-physiology index, *CPI* Composite physiologic index, *AEx* Acute exacerbation of idiopathic pulmonary fibrosis, *DSP* Distance-saturation-product, *CT* Computed tomography, *ILD* Interstitial lung disease, *HRCT* High-resolution computed tomography, *FVC* Forced vital capacity

The aims of this study were to re-evaluate a retrospective cohort of patients with IPF from Kuopio University Hospital (KUH), a tertiary hospital in eastern Finland, using the year 2011 international IPF guidelines [14]. In addition, we aimed to study the clinical factors that could differentiate between patient groups categorized according to their observed lifespan i.e. rapid, moderate and slow disease progression subgroup. We were interested in how accurately GAP and CPI as well as other clinical features and lung function parameters would be able to predict mortality in this retrospective IPF-cohort.

## Methods

### Patients and data collection

The study material has been thoroughly described in our previous studies [15, 16]. The study subjects were identified from medical records of KUH by using International Classification of Diseases version 10 (ICD-10) codes J84.1, J85.8 and J84.9 [17]. Two hundred twenty-three patients with pulmonary fibrosis (PF) treated in KUH between 1 January 2002 and 31 December 2012 were included into the initial evaluation and their clinical, radiological and histological information was collected [15, 16]. PF with a known etiology were excluded. Causes of death of the patients were obtained from the death certificates.

Smoking history was assessed as non-smoker, ex-smoker or current smoker [15]. Pulmonary function tests (PFT) were evaluated by applying the prevailing Finnish reference values [18]. The changes in PFT values at the 6 and 12 month time points were calculated as percentages. Radiological, clinical and histological data were re-analyzed according to the 2011 international guidelines for diagnosing IPF [14]. GAP stage was calculated using gender, age and PFT [4]. CPI was calculated from PFT results using formula from the original publication: 91.0 – (0.65 x % predicted DLco) – (0.53 x % predicted FVC) + (0.34 x % predicted FEV1) [3].

The patients were categorized into three groups according to their observed lifetime i.e. rapid (lifetime less than 2 years after diagnosis), moderate (lifetime 2–5 years after diagnosis) and slow (lifetime more than 5 years after diagnosis) as previously described [16]. The patients which were alive at the end of the study period with a follow-up time less than 5 years, were not included. Comparisons were also made between patients with a rapid disease course (survival less than 2 years) and patients with a slower course of disease (survival over 2 years) as well as between patients with a slow disease course (survival over 5 years) and patients with a more rapid course of disease (survival less than 5 years).

No consents for the inclusion into this retrospective study were collected, since the majority of the patients were already deceased (Finlex, The Data Protection Act 1050/2018 (4 and 6 §)) [19]. The study protocol was approved by the Research Ethics Committee of the Northern Savo Hospital District (statement 17/2013) and from the National Institute for Health and Welfare (Dnro THL/1052/5.05.01/2013). Permission to use data from death certificates was given by Statistics Finland (Dnro: TK-53-911-13). This study was conducted in compliance with the Declaration of Helsinki.

### Analysis

Group differences were examined by Kruskall-Wallis or Mann-Whitney U-test or by Chi-square test or Fisher exact test, when appropriate. Survival analysis was done using the Kaplan-Meier method with death and lung transplantation as end-points. Survival differences were compared using the log-rank test. Hazard analyses were calculated using Cox regression models. Cut-off values between groups with different disease courses were determined using ROC curve analysis. *P*-value < 0.05 was considered to be statistically significant. All data was analyzed using IBM SPSS Statistics version 21.

## Results

### Diagnosis and patient characteristics

A total of 132 patients with IPF were included in this study, 89 cases were excluded due to diagnoses other than IPF. The first HRCT was available in 131 (99.2%) patients but a second HRCT had been performed in only 66 (50.0%) patients. The mean time between the first and last HRCT scan was approximately 38 months. HRCT was not performed in one (0.8%) patient who had died suddenly. After the re-analysis of HRCT, 81 patients (61.8%) were classified as definite UIP, 29 (22.1%) as possible UIP, and 21 (16.0%) as non-definite UIP. Histological evidence of definite UIP was observed in 39 cases of which 22 had been designated as definite UIP, 4 possible UIP and 12 non-definite UIP on HRCT. One patient with histological evidence of definite UIP was not investigated by HRCT due to his sudden death. Nine out of the 21 patients categorized as non-definite UIP on HRCT, were cases with severe physical disabilities and comorbidities, which affected their possibilities for undergoing certain diagnostic procedures to histologically confirm their diagnosis. In these nine cases, that have all deceased, HRCT was categorized as non-definite UIP due to the distribution of honeycombing (*n* = 5), concomitant interference of heart failure (*n* = 3) or predominant emphysema (*n* = 1). However, re-examining all of the information of the course of disease and causes of death, these cases were categorized as IPF after careful consideration by the MDD. A total of 47 (35.6%) patients with possible UIP or non-definite UIP on HRCT were evaluated in the MDD to confirm their IPF diagnosis.

Patient characteristics of the whole cohort at baseline are presented in Table 2. In all, 73.5% of the patients were male and median survival was 42 months and 35.2% of the patients were non-smokers. Six patients were prescribed pirfenidone; of these, one patient discontinued the medication after 3 weeks due to severe gastrointestinal side effects. Three patients were prescribed nintedanib, two of which were previously treated with pirfenidone.

**Table 2:** Clinical characteristics of the cohort and patients with different courses of the disease

	Whole cohort	Rapid (R)	Moderate (M)	Slow (S)	*P*-value (R vs M)	*P*-value (R vs S)	*P*-value (M vs S)
N (%)^a^	132 (100)	40 (30.3)	37 (28.0)	39 (29.5)			
Male (%)	97 (73.5)	32 (80.0)	28 (75.7)	25 (64.1)	NS	NS	NS
Age - years (SD)	70.5 (9.80)	74.5 (8.6)	71.4 (9.3)	66.7 (11.0)	NS	0.003	NS
Median survival (Mo)	42.0	9.0	37.0	84.0	< 0.001	< 0.001	< 0.001
Non-smoker (%)	45 (34.1)	11 (31.6)	11 (31.4)	19 (48.7)	NS	NS	NS
Ex-smoker (%)	66 (50.0)	25 (67.6)	16 (40.0)	14 (35.9)	NS	0.025	NS
Current smoker (%)	17 (12.8)	1 (2.7)	8 (20.0)	6 (15.4)	0.012	NS	NS
FVC % predicted (SD)^b^	76.7 (18.5)	66.4 (18.0)	78.9 (18.8)	80.9 (16.1)	0.001	0.008	NS
DLco % predicted (SD)^c^	56.1 (17.5)	45.1 (15.7)	54.4 (13.8)	65.6 (19.3)	0.034	< 0.001	0.010
GAP I	67 (53.2)	9 (25.7)	17 (47.2)	31 (79.5)	NS	< 0.001	0.004
GAP II	47 (37.3)	14 (40.0)	19 (52.8)	8 (20.5)	NS	NS	0.004
GAP III	12 (9.7)	12 (34.3)			< 0.001	< 0.001	
CPI	39.9 (20.0)	49.0 (12.7)	40.3 (11.3)	33.4 (14.5)	0.020	< 0.001	NS
Definite UIP in HRCT	79 (61.7)	26 (70.3)	22 (55.0)	27 (69.2)	NS	NS	NS

^a^16 patients could not be assessed into any of the disease course groups (R, M, S) due to an inadequate follow-up time

^b^Spirometry results were missing from 6 patients – 5 from rapid and 1 from moderate group

^c^Diffusion capacity results were missing from 8 patients, all from the rapid group *R* Rapid, *M* Moderate, *S* Slow, *N* Number, *NS P*-value > 0.05, *SD* Standard deviation, *Mo* Months, *FVC* Forced vital capacity, *DLco* Diffusion capacity to carbon monoxide, *GAP* Gender-Age-Physiology index, *CPI* Composite physiologic index, *UIP* Usual interstitial pneumonia, *HRCT* High-resolution computed tomography

### Course of disease

Patient characteristics according to observed lifetime are presented in Table 2. The course of the disease of 40 (30.3%) patients was rapid, in 37 (28.0%) it was moderate and in 39 (29.5%) the course of the disease was slow. There were more ex-smokers in the rapid disease course group compared to the slow disease course group. In addition, there were fewer current smokers in the rapid disease course group than in the moderate group. DLco %, but not FVC %, at baseline differentiated significantly between the different disease course subgroups. There was no statistically significant difference in the proportion of definite UIP patterns in HRCT between the different disease course subgroups.

**Fig. 1:**
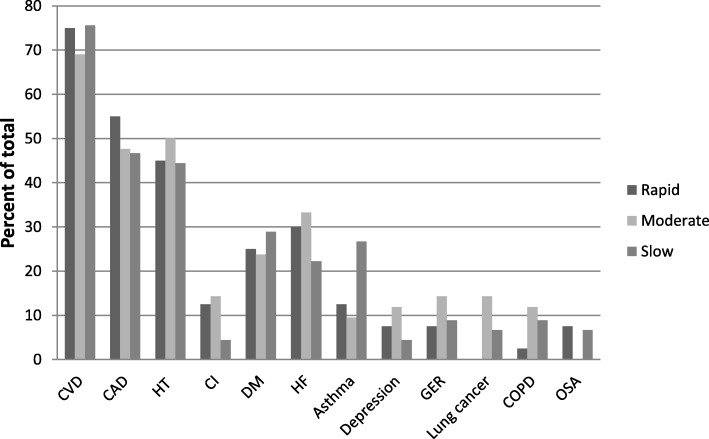
The most common comorbidities were cardiovascular diseases including coronary artery disease, hypertension and cerebral infarction. Patients with a rapid disease course (survival less than 2 years) had less lung cancer (0%) than patients with a moderate course of disease (survival 2–5 years) (14.3%, p = 0.026). Patients with a slow course of disease (survival more than 5 years) had more asthma (26.7%) than patients with shorter survival times (11.0%, *p* = 0.043). CVD, cardiovascular diseases; CAD; coronary artery disease; HT, hypertension; CI, cerebral infarction; DM, diabetes (types I and II); HF, heart failure for any reason; GER, gastroesophageal reflux; COPD, chronic obstructive pulmonary disease; OSA, obstructive sleep apnea

There were no significant differences in the numbers of comorbidities between different disease course subgroups. The most common comorbidities in all subgroups were cardiovascular diseases (CVD) (Fig. 1). Patients with a rapid disease course had less lung cancer (0%) than patients with a moderate disease course (14.3%, *p* = 0.026) whereas patients with slow disease course were more likely to suffer from asthma (26.7%) than patients with a more rapid course of disease (11.0%, *p* = 0.043) (Fig. 1). When the time-points of the comorbidity diagnoses were examined in the comparison of the different disease course subgroups, heart failure was more often diagnosed after the diagnosis of IPF in patients with a rapid disease course (30.0%) compared to patients with a slower course of disease (12.6%, *p* = 0.025). Patients with a slow course of disease had less often cerebral infarction (0%) after the diagnosis of IPF as compared to patients with a more rapid course of disease (11%, *p* = 0.026). Diabetes had also been more often diagnosed before the diagnosis of IPF in patients with a slow disease course (6.7%) as compared to patients with a more rapid course of disease (0%, *p* = 0.043).

When medications for comorbidities were compared between the groups, fewer patients with a slow course of disease (31.1%) were prescribed medications affecting platelet function including acetyl-salicylic acid, dipyridamole and clopidogrel, than patients with a more rapid disease course (51.2%, *p* = 0.040). Patients with a slow disease course also used inhalation steroids more commonly (17.8%) than patients with a rapid disease course (4.9%, *p* = 0.026). In addition, patients with a rapid disease course used more often allopurinol (7.5%) compared to patients with a slower course of disease (0%, *p* = 0.030).

### GAP and CPI

CPI was significantly higher in the rapid disease course group when compared to moderate and slow groups. However, CPI did not differ significantly between moderate and slow disease course groups. Almost 80% of the patients living more than 5 years were allocated to GAP stage I at baseline, in addition, all the 12 patients that were allocated to GAP stage III at baseline had a rapid disease course. However, 40% of patients with a rapid disease course were allocated to GAP stage II and 25% to GAP stage I at baseline. In ROC curve analyses GAP staging had a slightly better accuracy (0.67) than CPI (0.64) in predicting mortality in 2 years (Table 3). In addition, both GAP and CPI were significantly related to survival in univariate analyses and the best risk prediction model included age, gender and CPI (Tables 4 and 5). The GAP index was not tested in the multivariate analyses since it is calculated using scores from age, gender and PFTs.

**Table 3:** Factors distinguishing rapidly progressive disease (survival less than 2 years)

	Survival < 2 years	Survival > 2 years	*P*-value	Cut-off value	Sensitivity	Specificity	Accuracy
Age (Y)	74.5	68.9	0.008	72.5	0.62	0.68	0.70
FVC % predicted	66.4	80.0	0.001	73.5	0.63	0.64	0.64
DLco % predicted	45.1	60.1	< 0.001	54.5	0.72	0.65	0.66
CPI	49.0	36.7	< 0.001	41.9	0.72	0.61	0.64
GAP stage	> 2	1	< 0.001	> 1	0.74	0.64	0.67
FVC decline 12 mo (%)	−24.17	−4.99	0.003	−13.2	0.80	0.75	0.75
DLco decline 12 mo (%)	−33.3	−3.93	0.001	−22.2	0.80	0.85	0.84

The table presents the predictive sensitivity, specificity and AUC of clinical variables in their abilities to differentiate those IPF-patients that survived less than 2 years after diagnosis from those surviving over 2 years after diagnosis

*y* Years, *AUC* Area under the curve, *FVC* Forced vital capacity, *DLco* Diffusion capacity to carbon monoxide, *CPI* Composite physiologic index, *mo* Months, *IPF* Idiopathic pulmonary fibrosis

**Table 4:** Univariate analysis revealing the predictors of survival

Factor	HR	95% CI	*P*-value
Age	1.05	(1.02–1.07)	< 0.001
Gender			
Female	Ref		
Male	1.54	(1.00–2.37)	0.048
GAP I	Ref		
GAP II	2.55	(1.64–3.95)	< 0.001
GAP III	23.29	(10.52–51.57)	< 0.001
FVC % predicted	0.98	(0.97–0.99)	0.003
DLco % predicted	0.97	(0.96–0.98)	< 0.001
CPI	1.04	(1.02–1.06)	< 0.001
FVC % change 6 mo^a^	0.98	(0.96–1.00)	0.033
DLco % change 6 mo^b^	1.00	(0.98–1.01)	0.370
FVC % change 12 mo^c^	0.97	(0.96–0.99)	< 0.001
DLco % change 12 mo^d^	0.99	(0.98–1.00)	0.020

*HR* Hazard ratio, *CI* Confidence interval, *Ref* Reference group, *GAP* Gender-age-physiology stage, *FVC* Forced vital capacity, *DLco* Diffusion capacity to carbon monoxide, *CPI* Composite physiologic index, *mo* Months

^a^*N* = 81, ^b^*N* = 94, ^c^*N* = 70, ^d^*N* = 88

**Table 5:** Multivariate analyses for survival

Factor	HR	CI (95%)	*P*-value
Model containing gender, age, FVC % and DLco % at baseline			
Age	1.08	(1.05–1.10)	< 0.001
Gender			
Female	Ref		
Male	1.85	(1.14–2.99)	0.012
FVC % predicted	0.99	(0.97–1.00)	0.050
DLco % predicted	0.97	(0.96–0.98)	< 0.001
Model containing gender, age, FVC %,DLco % at baseline and FVC % and DLco % change in 6 months^a^			
Age	1.08	(1.05–1.12)	< 0.001
Gender			
Female	Ref		
Male	2.12	(1.09–4.09)	0.026
FVC % change in 6 mo	0.97	(0.95–1.00)	0.033
Model containing gender, age, FVC %,DLco % at baseline and FVC % and DLco % change in 6 months and in 12 months^a^			
Age	1.10	(1.03–1.17)	0.003
DLco % predicted	0.95	(0.92–0.99)	0.013
DLco % change in 12 mo	0.94	(0.91–0.98)	0.001
Model containing gender, age and CPI			
Age	1.07	(1.05–1.10)	< 0.001
Gender			
Female	Ref		
Male	2.03	(1.26–3.28)	0.004
CPI	1.05	(1.03–1.07)	< 0.001

*HR* Hazard ratio, *CI* Confidence interval, *Ref* Reference group, *FVC* Forced vital capacity, *DLco* Diffusion capacity to carbon monoxide, *CPI* Composite physiologic index

^a^Table presents only factors with *p*-value < 0.05

### Clinical factors in mortality prediction

In ROC curve analyses 12 months change in DLco % displayed the highest specificity, sensitivity and accuracy for predicting mortality in 2 years. In other variables, the predictive accuracy was less than 0.80. In the univariate analysis, gender, PFT results and the change in FVC % at both 6 and 12 months and DLco % change in 12 months were significantly related to survival (Table 4). Age remained a significant predictor of survival in all multivariate analyses and DLco % seemed to be better than FVC % in predicting the risk of death in this cohort (Table 5).

## Discussion

This study characterized the demographics and survival of the patients with IPF in eastern Finland. Since the cohort represents the patients treated between the years 2002 and 2012, most of the patients e.g. 125 patients were not receiving current treatment, namely nintedanib and pirfenidone. Thus the potential effect of these medications on disease progression can be considered as minimal. We evaluated retrospectively the clinical demographics and comorbidities when the individuals were categorized into the groups of rapidly, moderately and slowly progressive disease according to their survival times after diagnosis. In addition, GAP, CPI and single clinical factors were assessed in mortality prediction. Furthermore, cut-off values were calculated to assist in separating rapidly progressive disease group.

Our results on the characteristics of IPF are broadly consistent with previous studies with respect to age, gender distribution, PFT, survival and smoking history [10, 20, 21]. The patients were relatively uniformly divided into three disease course groups. Patients with a rapid disease course differed significantly from the more slowly progressive disease course groups in terms of age, smoking history, CPI values as well as PFT and PFT change in 12 months. Even though the rapid disease course group could be differentiated from other disease course groups at baseline in terms of clinical values, the predictive accuracy of any single factor remained generally less than 0.80. We observed, however, that age, gender and CPI as well as DLco %, but not FVC %, at the time of diagnosis were independently related to an increased risk of death. Conflicting results on the significance of age as a prognostic factor have been reported in earlier studies. King et al. presented that patients less than 50 years old lived longer than their older counterparts whereas in recent studies the age at the time of diagnosis has had no predictive value [21–23]. In contrast, another study revealed that in a multivariate analysis, age and PFT, but not gender, were significant predictors of survival [24].

More patients with moderately progressing disease belonged to GAP stage II and fewer to GAP stage I as compared to patients with the slowly progressing disease. This may at least partly be due to the lower DLco % in the moderate group, since gender distribution, age and FVC % did not differ between the slowly and moderately progressing subgroups. The mortality of rapid progressors was remarkably higher than that assessed by the GAP staging since 23 out of 40 patients experiencing a rapid progression had been categorized into GAP stages I and II, but not III. GAP staging and its association with increased risk of death were recently tested in a larger trial, showing that the risk of death was significantly increased for GAP stage III patients, but not for GAP stage II patients as compared to GAP stage I [25]. Similarly, in our study, hazard analyses revealed that GAP stages III and II patients had an increased risk of mortality as compared to GAP stage I patients. However, in the ROC curve analysis, only GAP stage III was predictive for 2-year mortality as compared to GAP stage I. The researchers of the original study introducing the GAP index and staging system proposed that patients belonging to GAP stage II should be listed for lung transplantation, if appropriate, and a recent study investigating GAP and CPI in patients undergoing a lung transplantation assessment revealed that CPI and GAP were better than single PFT values in predicting the mortality of the patients listed for lung transplant [4, 12]. The results of our study support this view since all patients of GAP stage III and as many as 40% of GAP stage II patients had a rapid course of disease i.e. dying in less than 2 years, a finding which indicates that the consideration of palliative care should not be delayed until GAP III as proposed previously [4].

The patients with the definite UIP pattern in HRCT have been reported to have poorer prognosis than patients with possible UIP and not-UIP patterns [26]. In our study, the proportion of definite UIP patterns, however, did not differ between disease progression subgroups and hence the radiological pattern was not related to prognosis. The results of our study were also in a similar direction to those of Yamauchi et al. i.e. honeycomb changes in HRCT were not related to prognosis [27].

A study using a similar division of disease course subgroups as applied here, reported that patients with a rapid disease course had less gastro-esophageal reflux and more diabetes and lung cancer than patients with a slow disease course, results that we could not confirm [28]. On the contrary, patients with a rapid disease course did not have lung cancer in our study cohort. However, similar to our results, they reported a higher percentage of males and patients with a smoking history in the rapid progression group in comparison with the other groups [28]. In our study cohort patients with a rapid disease course used more allopurinol, but allopurinol has not been related to increased risk of death in IPF [15, 29]. Hyldgaard et al. reported that the diagnosis of any CVD after the diagnosis of IPF was related to a poorer prognosis [30]. Instead, we observed that heart failure, but not CVDs, was diagnosed after the diagnosis of IPF more often in the rapid disease progression subgroup as compared to the other subgroups. However, the patients with a rapid disease course used more often drugs affecting platelet function than patients with a less rapid disease course, which can be considered to be a sign of more severely manifested CVDs in these patients dying relatively soon after diagnosis. Patients with a slow disease course were suffering from asthma and were more frequent users of inhaled steroids than patients with a rapidly progressing disease and it can be speculated that the use of inhaled steroids may slow disease progression in IPF.

CPI has been revealed to be a significant and independent predictor of 3-year survival; in that work, CPI value > 41 was the cut-off value [31]. In confirmation of those results, a CPI value over 42 was a predictor of 2-year mortality in our study cohort. In both the univariate and multivariate analyses, CPI was evaluated to be a significant predictor of survival. However, in the ROC analysis for 2-year mortality, CPI did not perform as well as GAP staging, DLco % predicted or FVC and DLco change in 12 months. Similar results have been published in a larger cohort revealing that DLco % was more accurate than CPI in predicting 12- and 24-month mortality, but in contrast to our results, CPI outperformed GAP staging in that study [10]. Also at odds with our results, a recently published study comparing CPI and GAP indicated that AUC was higher for CPI than GAP in 1-, 2- and 3-year mortality [5]. The difficulty encountered with GAP staging seems to be that GAP stages I and III predict low and high risk of mortality, respectively, but GAP stage II patients seem to have more an unpredictable course of disease. However, the researchers of the original GAP study recommended close monitoring of GAP II patients at 3 to 6 months intervals, a practice which seems highly advantageous in the light of our results.

The relatively small and retrospectively collected material can be considered as limitations of this study. It is possible that some patients with IPF have not been correctly coded into the hospital register and for that reason they were not included into this study. In addition, some patients may have been estimated to be too fragile to be subjected to diagnostic procedures and for that reason, the diagnosis of IPF was never established. Another limitation was that there was some missing information such as spirometry results from 6 cases, which meant that their GAP stage could not be calculated. This cohort included patients treated in KUH over a 10 year period between the years 2002 and 2012. Since the diagnostic criteria of IPF were updated in 2011, different diagnostic criteria were utilized during the study period. However, all patients originally gathered from hospital register using ICD-10 codes were meticulously re-assessed and re-classified using the year 2011 criteria, and the cases representing some other type of ILDs were excluded [14].

## Conclusions

Two risk prediction indexes, CPI and GAP, as well as multiple single clinical and physiological factors were tested in separate subgroups with strictly defined disease courses. We found that even in this well-characterized patient cohort, the prognostic value of single clinical factors remained low. CPI and GAP staging were useful in assessing the disease severity, but the accuracy of predicting 2-year mortality was slightly better for GAP than CPI. The majority of the patients experiencing a rapid disease course, however, could not be detected with GAP staging.

## Data Availability

The datasets generated and analyzed during the current study are not publicly available due to the relatively small population of eastern Finland i.e. we could not guarantee individuals’ anonymity as the data was collected in a detailed manner. The data is available from the corresponding author on reasonable request.

## Abbreviations

ATS: American Thoracic Society
AUC: Area under the curve
CPI: Composite physiologic index
CVD: Cardiovascular disease
DLco: Diffusion capacity of carbon monoxide
DLco/VA: Potential value of diffusion capacity per liter of lung volume
ERS: European Respiratory Society
FEV1: Forced expiratory volume in one second
FVC: Forced vital capacity
GAP: Gender- Age-Physiology Index
HRCT: High-resolution computed tomography
ICD-10: International Classification of Diseases version 10
ILD: Interstitial lung disease
IPF: Idiopathic pulmonary fibrosis
KUH: Kuopio University Hospital
MDD: Multidisciplinary discussion
mo: Months
PFT: Pulmonary function tests
ROC: Receiver operating characteristic
SLB: Surgical lung biopsy
UIP: Usual interstitial pneumonia
USA: United States of America
VATS: Video-assisted thoracoscopic surgery
y: Years
